# Stability of low back pain reporting over 8 years in a general population aged 40/41 years at base-line: data from three consecutive cross-sectional surveys

**DOI:** 10.1186/1471-2474-14-270

**Published:** 2013-09-21

**Authors:** Nadège Lemeunier, Charlotte Leboeuf-Yde, Per Kjaer, Olivier Gagey

**Affiliations:** 1 Complexité, Innovation et Activités Motrices et Sportives, Bâtiment 335, UFR STAPS, Université d’Orsay Paris Sud 11, Orsay Cédex 91405, France; 2 Institut Franco-Européen de Chiropraxie, 72 Chemin de la Flambère, Toulouse 31300, France; 3 Bicêtre University Hospital, AH-HP Paris, F-94270, JE-2494 Université paris-Sud, Orsay F-91405, France; 4 Research Department, Spine Center of Southern Denmark, Hospital Lillebaelt and Institute of Regional Health Services Research, Clinical Locomotion Network, University of Southern Denmark, Ostre Hougvej 55, Middelfart, Denmark; 5 Institute of Sports Science and Clinical Biomechanics, Clinical Locomotion Network, University of Southern Denmark, Odense, Denmark

**Keywords:** Epidemiology, General population, Cohort, Cross-sectional study, Prevalence, Low back pain, Trajectory, Non-responders

## Abstract

**Background:**

A recent review on the natural course of low back pain (LBP) in the general population indicated that the LBP reporting pattern is fairly constant over time. Furthermore, the LBP status at baseline (yes/no) seems to be predictive of the future course. When fluctuations occur, they seem most common between the nearest categories. However, in the majority of articles, non-responders were not taken into account in the analyses or interpretation of data, meaning that estimates may have been biased. Further, all reviewed studies included study participants of many different age groups. Data from three cross-sectional surveys over 8 years of the same cohort made it possible to answer the following questions: 1) Would the prevalence estimates of LBP be stable over time? 2) How would results change when taking into account non-responders? 3) Is the LBP reporting over the three survey periods stable at an individual level, taking into account also the non-responding group?

**Methods:**

Data from three subsequent cross-sectional surveys of a study sample were available and questions about LBP were asked at baseline and also 4 and 8 years later. Study participants were 40/41 years at base-line and initially randomly selected from the general Danish population. Data were analyzed with STATA/IC 12, and presented with percentages and 95% confidence intervals.

**Results:**

The majority of participants reported to have had LBP in the preceding year but not having taken sick leave in relation to this pain. LBP was stable or relatively stable for the study participants as they progressed through their fifth decade. This was true on a population basis and also on an individual level. When non-responders were taken into account the results did not change.

**Conclusions:**

This study confirmed the results from our recent review; both presence and absence of LBP seem to be predictive for the future course. The percentage of non-responders in this type of study may not be as important as previously thought in relation to the presence/absence of LBP.

## Background

Low back pain (LBP) is a wide-spread condition in the general population with an annual prevalence in many studies shown to be at least 50% [1]. It is difficult to diagnose [2], and because the causes are not well understood it is also difficult to treat and to prevent.

Previously, LBP was considered a disease with spontaneous cure and it was classified according to the anatomical location and duration of the pain (i.e. acute, sub acute or chronic LBP) [3]. Nowadays, it is becoming increasingly clear that LBP is more of a recurring or chronic condition, both in clinical [4,5] and non-clinical populations [6], with a fluctuating course over time [7]. However, not much is known of this fluctuating pattern.

A recent review of eight studies on the natural course of LBP in the general population indicated that the LBP reporting pattern (i.e. LBP yes/no) is fairly constant regardless whether people are surveyed at short or long intervals, many times per year or with several years’ intervals and also regardless if the study population is a general population or a working population [6]. In other words, those who have LBP at the onset of the observation period are likely to report it again at subsequent surveys and those without LBP keep on reporting none. When fluctuations occur between studies, they seem most common between neighboring categories, so that those with some LBP in one survey may develop into having more or none in a subsequent survey, but rarely will those without LBP develop persistent or severe LBP nor will those with persistent of severe LBP recover so as to report none [8-11].

Although the findings in the aforementioned review were consistent, it was noted that in all studies age groups were mixed, which might hide subgroup differences in relation to age, if persistence of LBP is age-dependent. Further, in all studies but one, non-responders were not taken into account but simply removed from the analyses, or at least from the interpretation of data, meaning that estimates of LBP may have been inflated or deflated in a non-transparent manner depending on the profiles and sizes of the groups of drop-out subjects. The question of how constant LBP is in the general population is important from a public health perspective. It is therefore relevant to look into this issue in some more detail.

We had access to data on LBP from three consecutive surveys of a study sample which was 40/41-years at base-line, initially randomly selected from the general Danish population, in which questions had been asked about their LBP also 4 and 8 years later. These data made it possible to obtain answers to the following questions:

1. Would the prevalence estimates of LBP be stable over time, in study subjects aged 40/41 at base-line, and aged 44/45 and 48/49 at the two subsequent follow-ups?

2. How would results change when taking into account non-responders?

3. Was the LBP reporting over the three survey periods stable at an individual level, taking into account also the non-responding group?

However, because not all study subjects participated at each survey, we also considered if the three overlapping samples were similar over time in relation to gender, employment status and educational level, presence or not of LBP past year, and numbers of days with LBP and sick leave in the preceding year.

## Methods

### Design, brief description of the study, and ethics approval

This report is based on a secondary analysis of data from three consecutive cross-sectional surveys on the same cohort, a sample from the Danish general population. The first survey was conducted in 2000/01 and the others again 4 and 8 years later.

The Danish Regional Committee of Ethics approved this project (N°20000042 and 96/272) and the database was approved by the Danish Data Protection Agency (N°2000-53-0037).

### Description of the initial study sample

**Figure 1:**
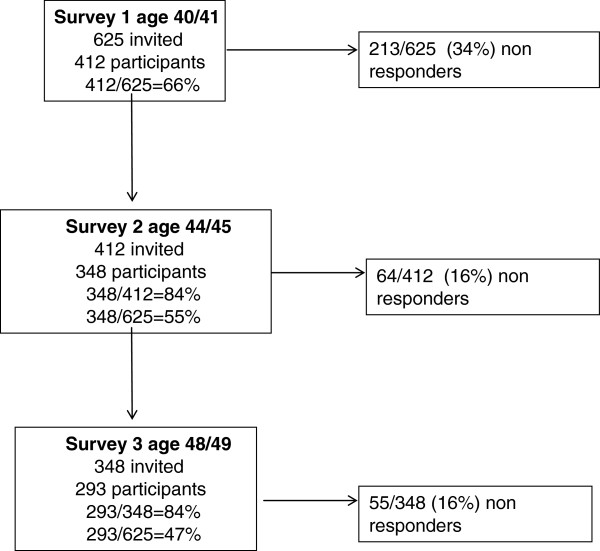
Flow-chart showing the study sample sizes and response rates at three subsequent surveys.

In 2001, 625 people aged 40/41, living in the county of Funen (Denmark) were randomly chosen by the Central office of Civil Registration with the aim of being representative of the general Danish population. Of these, 412 (66%) participated in survey 1 and the response rate was 84% (348/412 and 293/348), in both survey 2 and survey 3. Please, see flow-chart Figure 1.

The study sample at base-line was previously shown to be only slightly different from the Danish general population on the level of education and the employment status variables [12]. People with basic school and vocational education/training were slightly under-represented while the short and medium levels of education were slightly over-represented [12].

### Data collection

At baseline, questions were asked on employment status and education, lifestyle and the history of LBP and sick leave. The LBP questionnaire was previously validated in the Danish population for reliability and reproducibility [13,14]. The questions about LBP and sick leave were identical at each survey. Participants were also given a physical examination and an MRI examination.

At the two follow-up surveys, participants filled in a questionnaire at the research site, followed by an MRI scan. In this article, data on LBP contained in the three questionnaires were used.

Included in the questionnaires were two questions on LBP in the preceding year (“Have you in the past year been bothered by your low back?” and “For how many days have you been bothered by your lower back within the past year?”) and one on sick-leave (“For how many days have you been on sick leave because of low back trouble within the preceding year?”). The question on number of days with LBP was used as the outcome variable.

### Variables of interest

From the base-line questionnaire, the following background variables were taken into account:

– Sex

– Six types of employment status: self employed, assisting spouse (i.e. assisting self-employed partner), employed, unemployed, pensioners and others outside the labor force

– Six levels of education: basic school, general upper-secondary education, vocational education/training, short-cycle higher education, medium-cycle higher education, and long-cycle higher education.

The following two variables were used from each of the three surveys:

– LBP in the past year (yes, no) and total number of days with LBP in the past year (0, 1–30, >30 days)

– Total number of days with sick-leave because of LBP in the past year (0, 1–30, >30 days; yes/no).

The outcome variable (LBP in the past year) was classified into 0, 1–30 and >30 days in accordance with previous Danish epidemiologic back pain studies [8,15-23].

### Data management and analysis

The analysis of data was divided into three stages: 1) Description of the study samples, 2) Representativeness at the subsequent surveys, and 3) Research questions.

1) A description of the study sample at base-line was made with the background, LBP and sick-leave variables. Further, the number of days with sick-leave in the past year in relation to the number of days with LBP in the preceding 12 months was cross-tabulated at each survey, in order to further describe the study sample.

2) Comparisons were made for the same descriptive variables between responders and non-responders at the second and the third surveys.

3) To obtain answers to the research questions, the non-responders at survey 2 and survey 3 had to be taken into account.

a. The LBP prevalence estimates were therefore calculated at each survey using three different methods.

i. Prevalence estimates of LBP in surveys are calculated on the basis of the people participating in that survey. However, in follow-up surveys, there is often a considerable group of non-responders, who are usually ignored [6]. We called this the “usual” method and calculated the prevalence estimate at the second and third surveys based on the number of participants at that respective survey, i.e. x persons with LBP divided by all the participants at the relevant survey (multiplied by 100).

ii. The second method was based on the assumption that LBP does not change much over time [6]. To calculate the prevalence estimates in surveys 2 and 3, we therefore used the “same as before” method. When calculating the prevalence estimates, the non-responders in a given survey were therefore allocated to the same category as where they were found in the preceding survey.

iii. The third method was based on the assumption that the non-responders were likely to have moved into the worst category (>30 days). The reason for this assumption was that we considered it possible that the most disadvantaged and ill were most likely to become non-responders in a survey of this type. When calculating the prevalence estimates in surveys 2 and 3, non-responders were therefore allocated to the category of >30 days.

b. To study the individual course of days with LBP over the period of the three surveys, cross tabulations were made for survey 1 vs. survey 2, and survey 2 vs. survey 3, showing also the relative size of the non-responders. The purpose of these analyses was to identify the most common patterns of change over time. This transition was described as “stayed in same category” and “moved to another category”. If there was a move to another category, it was described to which one.

All data were analyzed with STATA/IC 12. Percentages and 95% confidence intervals [CI] were provided. Percentages were rounded up to the nearest whole figure, hence percentages do not always add up to 100.

## Results

### Description of study sample

As this is a secondary analysis, information on sample sizes and response rates were given in the Methods section but is provided also in Figure 1. The majority of participants were female (52, 54 and 54% at each survey, respectively). LBP in the preceding year was reported by almost 70% at the three surveys. In all three surveys, sick-leave had not been taken in the preceding year in the majority of cases (approximately 80%) (Table 1). Over the three surveys, of those with 0 days of LBP, 1-2% reported to have taken sick-leave because of LBP in the preceding year. Among those with 1–30 days of LBP past year, 15% - 18% had taken some sick-leave, usually for 1–30 days, whereas 29%-47% of those with LBP >30 days had taken some sick-leave, mainly either 1–30 days or > 30 days (detailed data available from the authors on request).

**Table 1:** Comparison of social factors, low back pain (LBP) and sickleave obtained at baseline for responders and non-responders at three subsequent surveys (Survey 1, survey 2 and survey 3)

**Variables of interest**	**Survey 1**		**Survey 2**		**Survey 3**	
	**Responders% [CI] (n = 412)**	**Non responders% [CI] (n = 213)**	**Responders% [CI] (n = 348)**	**Non responders% [CI] (n = 64)**	**Responders% [CI] (n = 293)**	**Non responders% [CI] (n = 119)**
**Gender**						
Female	52 [47–57]	45 [38–52]	54 [49–59]	41 [29–53]	54 [48–60]	47 [38–56]
**Employment status**						
Self employed	7 [5–9]	-	7 [4–10]	8 [1–15]	7 [4–10]	8 [3–13]
Assisting spouse	0	-	1 [0–2]	0	0	1 [0–3]
Employed	84 [80–88]	-	86 [82–90]	**72** [61–83]	87 [83–91]	**76** [68–84]
Unemployed	4 [2–6]	-	3 [1–5]	**9** [2–16]	3 [1–5]	**8** [3–13]
Pensioner	3 [1–5]	-	2 [1–3]	**8** [1–15]	1 [0–2]	**6** [2–10]
Others outside labor force	2 [1–3]	-	2 [1–3]	3 [0–7]	2 [0–4]	3 [0–6]
**Highest educational level**						
Basic school	22 [18–26]	-	21 [17–25]	**28** [17–39]	19 [15–23]	**30** [22–38]
General upper-secondary education	2 [1–3]	-	3 [1–5]	0	2 [0–4]	3 [0–6]
Vocational education/training	31 [27–35]	-	32 [27–37]	**25** [14–36]	34 [29–39]	**24** [16–32]
Short-cycle higher education	20 [16–24]	-	21 [17–25]	17 [8–26]	21 [16–26]	19 [12–26]
Medium-cycle higher education	19 [15–23]	-	18 [14–22]	23 [13–33]	19 [15–23]	18 [11–25]
Long-cycle higher education	6 [4–8]	-	5 [3–7]	6 [0–12]	5 [3–7]	7 [2–12]
**LBP past year**						
Yes	69 [65–73]	-	68 [63–73]	**77** [67–87]	69 [64–74]	**76** [68–84]
**Number of days with LBP past year**						
0	30 [26–34]	-	32 [27–37]	**20** [10–30]	32 [27–37]	**24** [16–32]
1-30	45 [40–50]	-	45 [40–50]	47 [35–59]	46 [40–52]	43 [34–52]
>30	25 [21–29]	-	23 [19–27]	**33** [21–45]	21 [19–24]	**34** [25–43]
**Sick-leave past year because of LBP**						
No	80 [76–84]	-	84 [80–88]	80 [70–90]	85 [81–89]	**74** [66–82]
**Number of days with sick-leave past year**						
0	80 [76–84]	-	80 [76–84]	80 [70–90]	82 [78–86]	74 [66–82]
1-30	15 [12–18]	-	15 [11–19]	13 [5–21]	13 [9–17]	18 [11–25]
>30	5 [3–7]	-	5 [3–7]	8 [1–15]	4 [2–6]	8 [3–13]

Values among non-responders at survey 2 and survey 3 that deviate the most from the baseline estimates of the responders are identified in bold.

### Comparison responders/non responders

A comparison of the final study sample in the first survey and the target population was reported in the Methods section. As seen in Table 1, there were also no big differences between responders and non-responders at the second and third surveys. However, employment status and educational level profiles became somewhat different at the second and third surveys as compared to the first. Those with LBP, particularly those with more than 30 days, and those who reported to have had LBP related sick-leave tended to become non-responders more frequently than the others.

### The one year period prevalence estimates

With the “usual method” of calculating prevalence estimates, these were found to be stable over time. LBP for 1 to 30 days was most commonly reported whereas LBP > 30 days was found in about one quarter, at all three surveys (Table 2, columns 2, 3 and 6).

**Table 2:** Days with low back pain (LBP) in the preceding year in a Danish general population aged 40/41 at baseline surveyed 3 times over 8 years

	**Survey 1**	**Survey 2**			**Survey 3**		
	**Including the responders only**	**Including the responders only**	**SAME AS BEFORE SCENARIO: Including the non responders categorized under their previous sub-group**	**WORST CASE SCENARIO: Including the non responders categorized under the worst sub-group (>30)**	**Including the responders only**	**SAME AS BEFORE SCENARIO: Including the non responders categorized under their previous sub-group**	**WORST CASE SCENARIO: Including the non responders categorized under the worst sub-group (>30)**
	**N = 412**	**N = 348**	**N = 412**	**N = 412**	**N = 293**	**N = 348**	**N = 348**
**Number of days with LBP past year**	**% [95% CI]**	**% [95% CI]**	**% [95% CI]**	**% [95% CI]**	**% [95% CI]**	**% [95% CI]**	**% [95% CI]**
**0**	30 [26–34]	32 [27–37]	30 [26–34]	27 [23–31]	25 [20–30]	25 [20–30]	21 [17–25]
**1-30**	45 [40–50]	42 [37–47]	43 [38–48]	35 [30–40]	52 [46–58]	50 [45–55]	44 [39–49]
**>30**	25 [21–29]	26 [21–31]	27 [23–31]	37 [32–42]	24 [19–29]	25 [20–30]	36 [31–41]

Prevalence estimates have been calculated in 3 different ways: including 1) the responders only; 2) the non responders categorized under their previous sub-group; and 3) the non responders categorized under the worst sub-group (>30 days).

If the non-responders would carry forward their “preceding” LBP status or if they would move into the “worst” category (>30 days), no changes would occur to the prevalence rates calculated the first time (Table 2, columns 4 and 7; and columns 5 and 8, respectively).

However, the true prevalence of LBP in this population is not known because 34% of the target sample failed to participate in the study at base-line and the percentages of non-responders of the remaining sample in the second and third surveys were 15% and 29%, respectively, when calculated from the base-line survey sample.

### Individual course

Cross tabulations of the LBP variables between the surveys showed the individual transition of LBP over the study period for survey 1 vs. survey 2 (Table 3) and survey 2 vs. survey 3 (Table 4).

**Table 3:** LBP in a Danish general population at ages 40/41 (survey 1) and at 44/45 (survey 2)

**Number of days with LBP in the preceding year at survey 1 (N = 412)**	**Number of days with LBP in the preceding year at survey 2 in relation to survey 1 (N = 348 participants +64 non-responders) % [95% CI]**			
	**0**	**1-30**	**>30**	**Non-responders**
	**(n = 112)**	**(n = 146)**	**(n = 90)**	**(n = 64)**
**0** (n = 123)	44 [35–53]	38 [29–47]	7 [2–12]	11 [5–17]
**1-30** (n = 187)	24 [18–30]	44 [37–51]	16 [11–21]	16 [11–21]
**>30** (n = 102)	14 [7–21]	16 [9–23]	50 [40–60]	21 [13–29]

Cross tabulation of the number of days with LBP in the past year for survey 1 vs. survey 2 when taking non-responders into account. The table shows the proportions of people in each category in percentages (%) with 95% confidence intervals (95% CI) which were found in the same or in a different category of LBP duration at the 2nd survey as compared to the 1st survey.

**Table 4:** LBP in a Danish general population at ages 44/45 (survey 2) and at 48/49 (survey 3)

**Number of days with LBP in the preceding year at survey 2 (n = 348)**	**Number of days with LBP in the preceding year at survey 3 in relation to survey 2 (N = 293 participants +119 non-responders) % [95% CI]**			
	**0**	**1-30**	**>30**	**Non-responders**
	**(n = 72)**	**(n = 152)**	**(n = 69)**	**(n = 55)**
**0** (n = 112)	38 [29–47]	35 [26–44]	8 [3–13]	20 [13–17]
**1-30** (n = 146)	16 [10–22]	58 [50–66]	16 [10–22]	14 [8–20]
**>30** (n = 90)	8 [2–14]	31 [21–41]	40 [30–50]	19 [13–29]

Cross tabulations of the number of days with LBP in the past year for survey 2 vs. survey 3 when taking non responders into account. The table shows the proportions of people in each category in percentages (%) with 95% confidence intervals (95% CI) which were found in the same or in a different category of LBP duration at the 3rd survey as compared to the 2nd survey.

For both tables, the most common finding in each row is to remain in the same category over time. The second most common finding is to move up or down to one of the neighboring categories. For those with more than 30 days, though, at the second survey, the most common move was to exit the study.

## Discussion

To our knowledge, this is the first epidemiologic follow-up study in the general population, in which the transition of LBP was investigated for individuals born the same year. Identical LBP questions were asked three times in a row over a period of eight years and an identical definition of LBP was provided for the participants at each survey. The outcome variable has been extensively used and validated previously.

The results confirm what others have found [6], namely that the self-reported duration of LBP is fairly stable on a population basis. According to the past literature, this seems to be the case regardless how LBP is defined and how often and at what interval it is surveyed [6]. In our case, LBP was categorized in relation to its total duration in the preceding year; 0, 1–30, and >30 days.

Further, LBP fluctuates between surveys, usually by moving only one category up or down but not from extreme to extreme, again, confirming results indicated in previous research [6] that LBP is a stable condition also on an individual level. In particular, two similar studies to ours were identified [8,9], both performing three surveys over a period of 5 and 9 years, respectively but on people of varying age. Their outcome variable was also number of days with LBP in the preceding year. Their data included information on stability of both absence and presence of LBP or fluctuations between “neighboring” categories, as in our own study. Their results concur with ours.

In epidemiologic research, sampling method, sample size and response rates are important issues, as they may have an influence on the representativeness of the final study sample and hence on the external validity of the results. Unfortunately, it is difficult to motivate people from the general population to participate in studies, making high response rates hard to achieve, in particular over a series of surveys over a prolonged period of time carried out on the same cohort. For example, according to our previous review that included epidemiologic studies of the general population, the real response rates at the last follow-up ranged between 21% and 76%, when taking into account the participants at the first survey [6]. The consequences of this can be difficult to ascertain and are only rarely addressed in studies similar to this one.

Our response rates seemed high, when calculated as it is usually done, based on the number of people participating at each survey in relation to the participants at the previous survey. Nevertheless, these percentages would diminish, as is usually the case also in other studies, if the entire target sample were to be taken into account when calculating response rates. We therefore looked closer at the proportion of non-responders throughout our three surveys and noted that non-response was particularly common at base-line (approximately 1/3 of those invited were missing), meaning that already the initial study sample may be biased in some (unknown) way. At survey 2, 1/6 of the remaining sample disappeared and then again at survey 3 an additional 1/6, resulting in, at survey 3, a remaining group consisting of approximately half of those invited at the very beginning. This resulted in some differences between responders and non-responders, providing a hint on how to interpret the final data. We therefore found it relevant to demonstrate the size of the non-responder groups in our result tables.

In relation to the LBP variable, differences between responders and non-responders at the two follow-ups were noticed with relatively more losses to follow-up among those with LBP > 30 days than in the other two categories. Interestingly, though, the proportions of people who dropped out of our study were similar for each base-line category of LBP (11%- 21% and 14% – 21% at surveys 2 and 3, respectively). As, on an individual level, the stability of LBP reporting over time was high, the estimates would therefore not change much over time, assuming as we did that non-responders would remain where they were at the previous survey. Thus, the stable nature of LBP may make response rates less important than generally presumed, provided that the proportion of drop-outs is evenly distributed among the initial subgroups. However, this would be better understood with more detailed information on the nature of LBP in relation to severity and consequences.

From a research perspective, it would be relevant to find out if this constancy is stable also over shorter periods and if it covers different subgroups with different courses on a more short-term basis. Further, it would be necessary to investigate if summary subgroups, such as ours (0 days, 1–30 days and >30 days) really exist. How easily can one recall if the pain lasted 30 or 31 days? Can those with only a few days of pain really remember this or are they more likely to think they had had no LBP at all? Frequent data collection, such as using text messages or internet diaries, would be relevant, finally, to be able to validate this subgroup classification. Our next article will cover this issue.

## Conclusions

In conclusion, LBP is a stable or relatively stable condition for individuals in the general Danish population as they progress through their fifth decade. For patients and clinicians, this is important information as it indicates that presence of LBP, once there, is a more or less normal state and that absence of LBP probably indicates a degree of “immunity” to this condition. This statement is supported, not only by our recent review of studies from the general population [6] but also by a recent review of the one-year clinical course of non-specific LBP, which concluded that non-recovery is more common than recovery [24]. It is interesting that this phenomenon is so similar both in clinical and non-clinical populations, perhaps indicating that the clinical and natural courses are more or less identical.

## Competing interest

The authors declare that they have no competing interest.

## Authors’ contributions

PK was responsible for the epidemiologic study that formed the basis for this work. CLY, OG and NL formulated the research questions. PK and NL performed the analysis. All the authors were involved in interpreting the data, writing the manuscript and approving the final version.

## Pre-publication history

The pre-publication history for this paper can be accessed here:

http://www.biomedcentral.com/1471-2474/14/270/prepub

## References

[B1] Lebœuf-Yde C Fejer R Nielsen J OKyvik K Hartvigsen J Consequences of spinal pain: Do age and gender matter? A cross-sectional population-based study of 34 902 individuals 20–71 years of age BMC Musculoskelet Disord 2011 12 39 21299908 10.1186/1471-2474-12-39 PMC3041727

[B2] Deyo R Rainville J Kent D What can the history and physical examination tell us about low back pain? JAMA 1992 6 760 1386391

[B3] Spitzer WO LeBlanc RE Dupuis MS Scientific approach to the assessment and management of activity-related spinal disorders. In Monograph for Clinicians: Report of the Quebec Task Force on Spinal Disorders Spine 1987 12 s16 s21 2961086

[B4] van den Hoogen HJ Koes BW van Eijk JT Bouter LM Deville W On the course of low back pain in general practice: a one year follow up study Ann Rheum Dis 1998 57 1 13 19 9536816 10.1136/ard.57.1.13 PMC1752458

[B5] Stanton TRLJ Maher CG Hancock MJ How do we define the condition 'recurrent low back pain'? A systematic review Eur Spine J 2010 19 533 539 19921522 10.1007/s00586-009-1214-3 PMC2899839

[B6] Lemeunier N Lebœuf-Yde C Gagey O The natural course of low back pain: a systematic critical literature review Chiropr Man Therap 2012 20 1 33 10.1186/2045-709X-20-33 PMC3599187 23075327

[B7] Tamcan O Mannion AF Eisenring C Horisberger B Elfering A Müller U The course of chronic and recurrent low back pain in the general population Pain 2010 150 3 451 457 20591572 10.1016/j.pain.2010.05.019

[B8] Hestbæk L Lebœuf-Yde C Engberg M Lauritzen T Bruun NH Manniche C The course of low back pain in a general population. Results from a 5-year prospective study J Manipulative Physiol Ther 2003 26 4 213 219 12750654 10.1016/s0161-4754(03)00006-x

[B9] Maul I Läubli T Klipstein A Krueger H Course of low back pain among nurses: a longitudinal study across eight years Occup Environ Med 2003 60 497 503 12819283 10.1136/oem.60.7.497 PMC1740571

[B10] van Oostrom SH Verschuren VMM de Vet HCW Picavet HS Ten year course of low back pain in an adult population-based cohort - The Doetinchem Cohort Study Eur J Pain 2011 15 993 998 21429779 10.1016/j.ejpain.2011.02.007

[B11] Videman T Ojajärvi A Riihimäki H Troup JDH Low back pain among nurses Spine 2005 30 20 2334 2341 16227898 10.1097/01.brs.0000182107.14355.ca

[B12] Kjaer P Lebœuf-Yde C Korsholm L Sorensen JS Bendix T Magnetic resonance imaging and low back pain in adults: a diagnostic imaging study of 40-year-old men and women Spine 2005 30 10 1173 1180 15897832 10.1097/01.brs.0000162396.97739.76

[B13] Biering-Sørensen F A one year prospective study of low back trouble in a general population. The prognostic value of low back history and physical measurement Dan Med Bull 1984 31 362 375 6239755

[B14] Kuorinka I Jonsson B Kilbom A Vinterberg H Biering-Sørensen F Andersson G Jørgensen K Standardized Nordic questionnaire for the analysis of musculoskeletal symptoms Appl Ergon 1987 18 233 237 15676628 10.1016/0003-6870(87)90010-x

[B15] Lebœuf-Yde C Yashin A Lauritzen T Does smoking cause low back pain? Results from a population-based study J Manipulative Physiol Ther 1996 19 99 108 9064317

[B16] Lebœuf-Yde C Klougart N Lauritzen T How common is low back pain in the Nordic population? Data from a recent study on a middle-aged general Danish population and four surveys previously conducted in the Nordic countries Spine 1996 21 1518 1526 8817778 10.1097/00007632-199607010-00005

[B17] Lebœuf-Yde C Lauritsen J Lauritzen T Why has the search for causes of low back pain largely been nonconclusive Spine 1997 22 877 881 9127921 10.1097/00007632-199704150-00010

[B18] Lebœuf-Yde C Ohm Kyvik K At what age does low back pain become a common problem? A study of 29,424 individuals aged 12–41 years Spine 1998 23 228 234 9474731 10.1097/00007632-199801150-00015

[B19] Lebœuf-Yde C Ohm Kyvik K Bruun H Low back pain and life-style. Part I: Smoking. Information from a population-based sample of 29,424 twins Spine 1998 23 2207 2214 9802163 10.1097/00007632-199810150-00012

[B20] Lebœuf-Yde C Ohm Kyvik K Bruun H Low back pain and life-style. Part II - Obesity. Information from a population-based sample of 29,424 twin subjects Spine 1999 24 779 784 10222529 10.1097/00007632-199904150-00009

[B21] Hartvigsen J Bakketeig LS Lebœuf-Yde C Engberg M Lauritzen T The association between physical workload and low back pain clouded by the “healthy worker” effect. Population-based cross-sectional and 5-year prospective questionnaire study Spine 2001 26 1788 1793 11493851 10.1097/00007632-200108150-00011

[B22] Hestbæk L Lebœuf-Yde C Kyvik KO Manniche C Is low back pain in youth associated with weight at birth? A cohort study of 8,000 Danish adolescents Dan Med Bull 2003 50 181 185 12812141

[B23] Hartvigsen J Kyvik KO Lebœuf-Yde C Lings S Bakketeig L Ambiguous relation between physical workload and low back pain: a twin control study Occup Environ Med 2003 60 109 114 12554838 10.1136/oem.60.2.109 PMC1740454

[B24] Itz CJ Geurts JW van Kleef M Nelemans P Clinical course of non-specific low back pain: a systematic review of prospective cohort studies set in care Eur J Pain 2013 17 1 5 15 22641374 10.1002/j.1532-2149.2012.00170.x

